# Porcine corneal cell culture models for studying epidemic keratoconjunctivitis

**Published:** 2013-03-20

**Authors:** Mirja Ramke, Elena Lam, Michael Meyer, Andreas Knipper, Albert Heim

**Affiliations:** 1 Institute of Virology, Hannover Medical School, Hannover, Germany; 2 Department of Ophthalmology, Hannover Medical School, Hannover, Germany; 3 Deutsche Gesellschaft für Gewebetransplantation (DGFG), Hannover, Germany

## Abstract

**Purpose:**

Epidemic keratoconjunctivitis (EKC) is a severe ocular infection caused by a few types (8, 19a [relabeled as 64 recently], 37, 53, and 54) of human adenoviruses (HAdVs). HAdVs are known for their strong host species specificity that limits studying HAdV virulence and pathophysiology in animal models.

**Methods:**

A HAdV infection model of primary porcine corneal epithelial cells (PPCE) and primary porcine corneal keratocytes (PPCK) was established and compared to primary human corneal epithelial cells (PHCE) and primary human corneal keratocytes (PHCK). Induction of interleukin-8 (IL-8) messenger RNA (mRNA), HAdV DNA replication, and the release of infectious virus progeny by the EKC-associated type HAdV-D37 and the non-EKC-associated type HAdV-D22 were studied.

**Results:**

PPCE and PPCK morphology and the expression of α2,3-linked sialic acid, the main receptor of EKC-associated HAdV types, were akin to human corneal cells (PHCE and PHCK). Induction of IL-8 mRNA was observed as early as 8 h after HAdV infection. Induction of IL-8 mRNA by HAdV-D37 infection was significantly higher (p≤0.001) than by HAdV-D22 infection in PPCE, PPCK, PHCE, and PHCK. Detection of HAdV-DNA replication, release of infectious virus progeny, and the development of cytopathic effect indicated that PPCE and PPCK were fully permissive for HAdV-D37 and HAdV-D22 replication as were the human corneal cells (PHCE and PHCK). Infectious virus titers after HAdV-D37 infection (1.0×10^5^ TCID_50_/ml) were significantly higher (p=0.001) than after HAdV-D22 infection (1.8×10^4^ TCID_50_/ml) in PPCE, PHCE, and PHCK but not significantly different in PPCK.

**Conclusions:**

Primary porcine epithelial cells and keratocytes are nonhuman corneal cell culture models fully permissive for HAdV infection. The models hold promise for studying the virulence and pathophysiology of EKC-associated adenovirus types compared to other adenovirus types.

## Introduction

Human adenoviruses (HAdV) are common pathogens with a wide range of tropism displayed by many types and are classified into seven species (A–G) comprising 65 types [1]. Epidemic keratoconjunctivitis (EKC) is caused typically by only three types, HAdV-D8, HAdV-D19a, and HAdV-D37 [2,3]. EKC is the only adenoviral disease with significant subepithelial corneal involvement and characterized by conjunctivitis, epithelial corneal erosions, and infiltration of leukocytes into the subepithelial corneal stroma. The leukocyte infiltration leads to photophobia and impaired vision for months to years following infection [4,5]. Currently, no effective antiviral treatment for EKC is available [6].

Previously, it was reported that EKC-associated HAdV types use α2,3-linked sialic acid as primary receptor [7-9] instead of the coxsackie-adenovirus receptor (CAR), which is used by most HAdV types. Probably, this ability to target α2,3-linked sialic acid as the primary receptor contributes to the unique ability of a few HAdV types to cause EKC [8].

The multifocal and superficial leukocyte infiltration of the corneal stroma suggested the significance of the immune system for the pathogenesis of EKC [6,10]. Previous studies demonstrated induction of interleukin-8 (IL-8) messenger ribonucleic acid (mRNA) as an initial response after binding of EKC-causing HAdV types to the cell [11,12].

In 2005, HAdV-D53 was isolated from a patient with EKC [13]. HAdV-D53 is a new EKC-associated HAdV type with a recombinant genome consisting of sequences derived from the non-EKC-type HAdV-D22 (coding for the neutralization determinant) and the EKC-associated types HAdV-D8 and HAdV-D37, which code for the binding sites to the primary cellular receptor (α2,3-linked sialic acid) and the secondary receptor [3]. In an EKC mouse model, injection of HAdV-D53 into the cornea induced an early immune response as demonstrated by increased IL-8 mRNA and keratitis after 4 days, whereas HAdV-D22 injection did not [3]. Moreover, another new recombinant HAdV type (D54) was described [14]. Recently, the EKC-associated genome type HAdV-D19a was renamed HAdV-D64 [1].

HAdVs, including all EKC types, are host species specific, and a complete viral replication cycle including release of infectious virus progeny cannot be observed in animal models as, for example, the cotton rat [15], the New Zealand White rabbit [16,17], the Hollander rabbit [18], and a mouse model [19]. In the mouse model, HAdV gene expression was observed, but release of infectious virus progeny was not detected, suggesting that the viral replication cycle is blocked at a late stage. Results for HAdV-D8 replication in the cotton rat EKC model were contradictory [15,20]. However, three recent publications described efficient HAdV replication of a few HAdV types (e.g., 1, 4, 5, and 17) in immortalized porcine cell lines and primary cell cultures of several organs and tissues [21-23]. Unfortunately, replication of EKC-associated HAdV types and porcine corneal cell culture has not yet been studied.

Therefore, we established a primary porcine corneal epithelial cell and keratocyte cell culture model for studying the pathogenicity of EKC-associated HAdV types. Here we describe the isolation and culture conditions of porcine corneal epithelial cells (PPCE) and keratocytes (PPCK). Expression of α2,3-linked sialic acid by these cells, infection with EKC-associated and non-EKC-types of HAdV, and induction of the chemokine IL-8 were compared to human corneal epithelial cells (PHCE) and human keratocytes (PHCK).

## Methods

### Isolation of human and porcine keratocytes

This study was conducted according to our institution’s guidelines and the Declaration of Helsinki. Porcine corneal keratocytes were isolated from porcine corneas (*Sus Scrofa domestica*, *Deutsche Landrasse*, at the age of 4–6 months), gained from an abattoir (Schlachthof Hannover GmbH, Hannover, Germany). Human keratocytes were isolated from the remaining cornea-scleral rims from corneas used for transplantation. Corneas were incubated for 10 min in trypsin (Biochrom, Berlin, Germany) at 37 °C, followed by mechanical removal of the epithelium and the endothelium. The corneal stroma was cut into sections 2 to 3 mm in diameter, submerged in Dulbecco's Modified Eagle Medium (DMEM)/Ham's F-12 (1:1 mixture; Biochrom, Berlin, Germany) supplemented with 10% fetal bovine serum (FBS), 2 mM L-Glutamine, 100 IU penicillin, 100 µg/ml streptomycin (Cytogen, Sinn, Germany), 50 µg/ml gentamycin (Invitrogen, Grand Island, NY), and 2 µg/ml fluconazole (B. Braun, Melsungen, Germany). After the keratocytes had proliferated from the corneal stroma onto the cell culture surface, tissue pieces were removed with a sterile forceps, and the cells were passaged by trypsinization.

### Isolation of corneal epithelial cells

Primary human and porcine corneal epithelial cells were isolated from the corneas according to the same protocol. The limbal epithelium was dissected directly below Bowman’s membrane and incubated for 150 min at 37 °C in DMEM/ Ham’s F12 medium containing 2.4 U/ml Dispase II (Sigma-Aldrich, Munich, Germany). Ten ml of 5 mM EDTA was added for 5 min to stop the digestion, followed by centrifugation for 10 min by 336 ×g. After washing twice with phosphate buffered saline (PBS; 2.67 mM potassium chloride [KCl], 1.47 mM potassium phosphate monobasic [KH_2_PO_4_], 137.93 mM sodium chloride [NaCl], 8.06 mM sodium phosphate dibasic [Na_2_HPO_4_-7H_2_O]), the cells were cultured in 25 cm^2^ flasks (Sarstedt, Nümbrecht, Germany) in DMEM/HAM’s F12 medium (Biochrom) supplemented with 10% fetal calf serum (FCS), 5 µg/ml insulin, 10 ng/ml epidermal growth factor (EGF), and 100 IU penicillin/ 100 µg/ml streptomycin (Cytogen), 50 µg/ml gentamycin (Invitrogen), and 2 µg/ml fluconazole (B. Braun).

### Virus stocks

HAdV-type D22 was gained from the American Type Culture Collection (ATCC, Manassas, VA). HAdV-D37 was isolated from the eye of a patient with EKC. Both types were propagated on A549 cells, harvested at 70%–80% cytopathic effect (CPE) with three freeze–thaw cycles, and stored at −80 °C. Infectious virus particles per ml were quantified with the tissue culture infectious dose 50% (TCID_50_) method on A549 cells.

### Adenovirus infection of epithelial cells and keratocytes

All cell culture systems were infected with HAdV when about 95% confluent monolayers were observed. Cell cultures were washed with PBS^-^ and infected with HAdV-D22 or D37 at a multiplicity of infection (moi) of 10 TCID_50_/cell for 1 h at 37 °C in Ham’s F12/DMEM (Biochrom). Cells were washed twice with PBS^-^ and cultured at 37 °C in cell culture medium for the indicated intervals. Intracellular HAdV DNA concentrations were quantified with real-time PCR at the indicated time points, and release of infectious virus progeny to the culture supernatant was quantified with the help of the TCID_50_ method. Cells were harvested by trypsinization for DNA or RNA isolation. Total RNA was purified using the QIAshredder and RNeasy Mini Kit (Qiagen, Hilden, Germany) according to the manufacturer`s instructions.

### Reverse transcription (complementary DNA synthesis)

Residual genomic DNA in RNA preparations was digested with DNase I (Ambion, Grand Island, NY) for 1 h at 37 °C according to the manufacturer’s instructions. Before heat inactivation of DNAse I for 10 min at 75 °C, 5 mM EDTA was added to the samples to avoid chemical scission. cDNA synthesis from 500 ng of DNase I digested RNA was performed using deoxynucleotide triphosphates (dNTPs, Promega, Madison, WI) and Oligo (dT)_18_ Primers (Fermentas, St. Leon-Rot, Germany) and Superscript II reverse transcriptase (Invitrogen) according to the manufacturer’s instructions.

### Quantitative polymerase chain reaction

Real-time PCR for cytokine determination was performed using the Brilliant SYBR Green QPCR kit (Agilent Technologies, La Jolla, CA) in an Mx-3000P instrument (Stratagene, Cedar Creek, TX). Specific primers for human IL-8 [24] and porcine IL-8 (forward: 5′-CTG CTT TCT GCA GCT CTC T-3′, reverse: 5′-CAG ACC TCT TTT CCA TTG-3′) were used. Melting-curve analysis was performed to verify the accuracy of the amplicons. In the initial experiments, the amplification efficiency was determined for all primer pairs analyzing a dilution series of control cDNA. Quantification of the human [25] and porcine glyceraldehyde-3-phosphate dehydrogenase housekeeping gene (forward: 5′-ATT CTC CAG TCA CCT GCT GC-3′, reverse: 5′-CTT CAA GGC TTC GGA GTT TGG-3′) was performed in parallel in every PCR. Relative gene expression levels to glyceraldehyde-3-phosphate dehydrogenase were calculated using Rest 2005 relative expression software (Corbett Research, Sydney, Australia [26]). Quantitative PCR for HAdV DNA was performed as described previously [27]. Briefly, Taqman PCR was carried out on the ABI 7500 Real Time Cycler (AppliedBiosystems, Carlsbad, CA) in a total reaction volume of 100 μl with the following primers (each 0.4 μM): 5'-GCC-ACG-GTG-GGG-TTT-CTA-AAC-TT-3' and 5'-GCC-CCA-GTG-GTC-TTA-CAT-GCA-CAT-C-3'; and the probe (0.5 μM) 5'-TGC-ACC-AGA-CCC-GGG-CTC-AGG-TAC-TCC-GA-3' labeled with FAM at the 5' end and TAMRA at the 3' end. All other reaction components were included in the QuantiTect Multiplex Mix System (Qiagen). Reaction conditions were denaturation at 95 °C for 15 min, amplification for 45 cycles with an annealing temperature of 55 °C for 20 sec, an elongation step of 65 °C for 60 sec followed by denaturation at 95° for 30 sec.

### Cytochemistry

Lectin staining of epithelial cells and keratocytes was performed by incubation with fluorescein isothiocyanate (FITC)-labeled *Maackia amurensis* lectin I (MAAI; Vector Laboratories, Burlingame, CA). Keratocytes were grown to 95% confluence on coverslips and coated with 10 µg collagen I, whereas corneal epithelial cells were grown on collagen I coated 0.33 cm^2^ polyester filters (0.4 µm pore size, Corning, Coster, NY). Cells were fixed in 3% paraformaldehyde (PFA) for 15 min, washed three times with PBS^-^, permeabilized with 0.1% Triton X-100 for 10 min, blocked with 3% bovine serum albumin for 1 h, and washed again three times. Cells were incubated with FITC-labeled MAA I (10 μg/ml) for 60 min at 4 °C in a humid chamber and subsequently washed three times again for 10 min. Finally, cells were mounted on glass slides using Mowiol (Sigma-Aldrich, St. Louis, MO) supplemented with 25 mg/ml 1,4-diazabicyclo(2,2,2)octan.

## Results

### Isolation and adherent culture of primary porcine and human corneal cells

**Figure 1:**
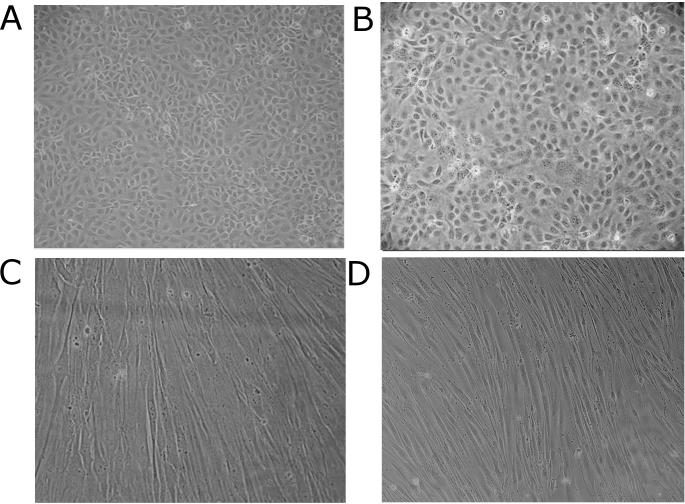
Phase contrast microscopy of primary porcine corneal epithelial cells (PPCE; **A**), primary human corneal epithelial cells (PHCE; **B**), primary porcine corneal keratocytes (PPCK; **C**), and primary human corneal keratocytes (PHCK; **D**).

Limbal corneal cell isolation from a single porcine cornea yielded 3–5×10^5^ PPCE compared to 1–3×10^3^ corneal epithelial cells from a human cornea (PHCE). Cells formed colonies after 2 to 3 days. The morphology of the PPCE resembled the uniform morphology of primary human epithelial cells (Figure 1A,B). PPCE can be maintained in culture for two passages (about 3 weeks) before they start to detach, compared to three passages by primary PHCE. However, the higher yield of PPCE and the abundance of porcine eyes made this cell culture model attractive for corneal epithelial infection studies in comparison to human epithelial cells.

Proliferation of primary keratocytes from small dissecates of porcine and human cornea stroma was observed after 7 days in culture medium. The morphology of the PPCK resembled the morphology of primary human corneal keratocytes (PHCK), and both had the typical fibroblastoid shape (Figure 1C,D).

### Alpha 2,3-linked sialic acid expression

**Figure 2:**
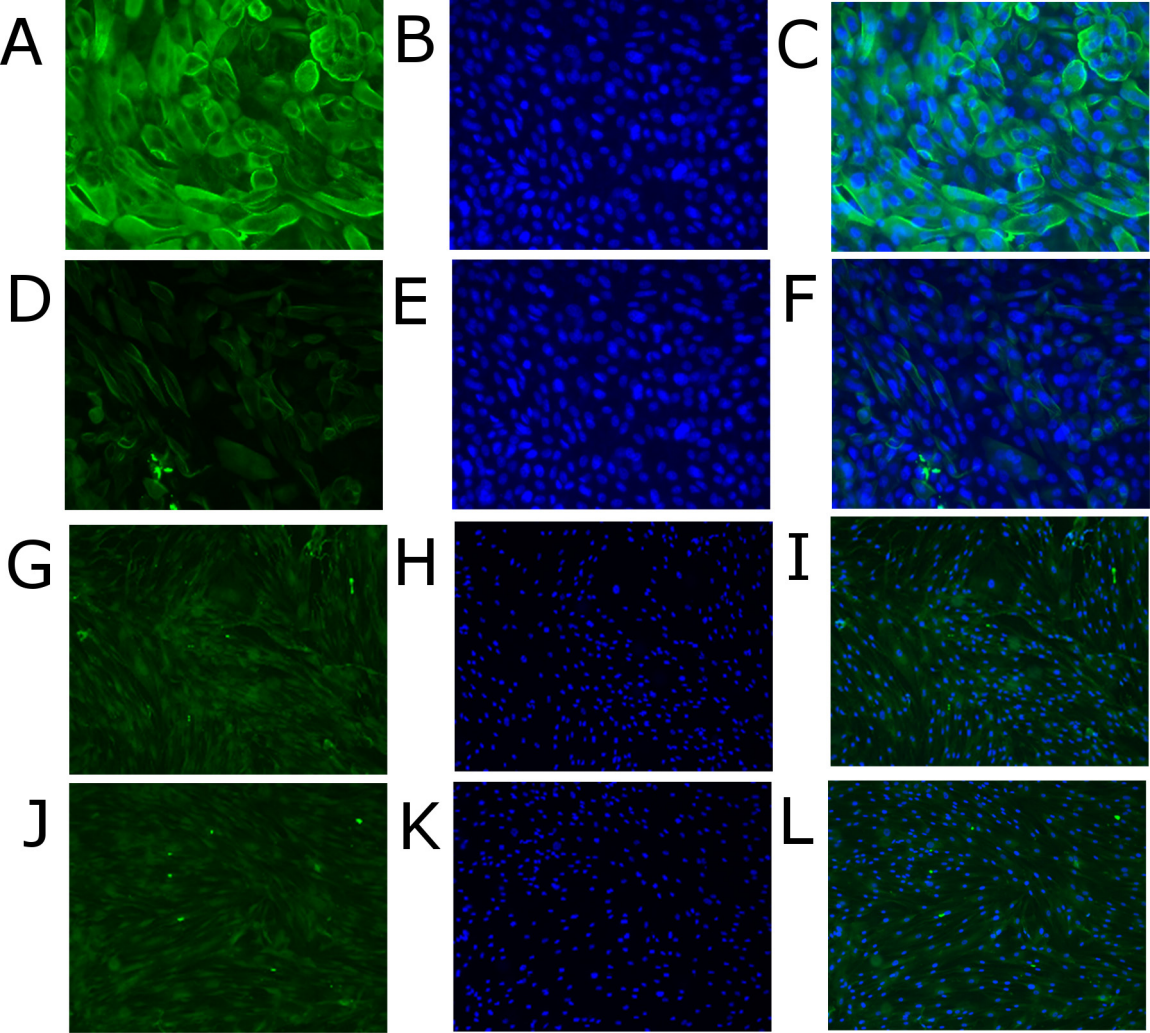
Comparison of porcine corneal epithelial cells (**A**–**C**) with primary human corneal epithelial cells (**D**–**F**), magnification 250X, and of primary porcine corneal keratocytes (**G**–**I**) with primary human corneal keratocytes (**J**–**L**), magnification 50X. Alpha 2,3 sialic acid terminal saccharides were stained with fluorescein isothiocyanate–labeled *Maackia amurensis I lectin* (green, **A**, **D**, **G**, **J**), and the nuclei were counterstained with 4', 6-diamidino-2-phenylindole (blue, **B**, **E**, **H**, **K**). **C**, **F**, **I**, and **L** show the merged images.

Porcine and human corneal cells expressed α2,3-linked sialic acid as indicated by staining with FITC-labeled *Maackia amurensis* lectin I. Alpha 2,3-linked sialic acid is the cellular receptor for EKC-causing HAdV types [8]. PPCE expressed more α2,3-linked sialic acid compared to human epithelial cells (PHCE; Figure 2A,B), whereas the α2,3-linked sialic acid expression levels of the porcine and human keratocytes were similar (Figure 2C,D).

### Replication of human adenovirus-D37 DNA in human and porcine corneal cells

**Figure 3:**
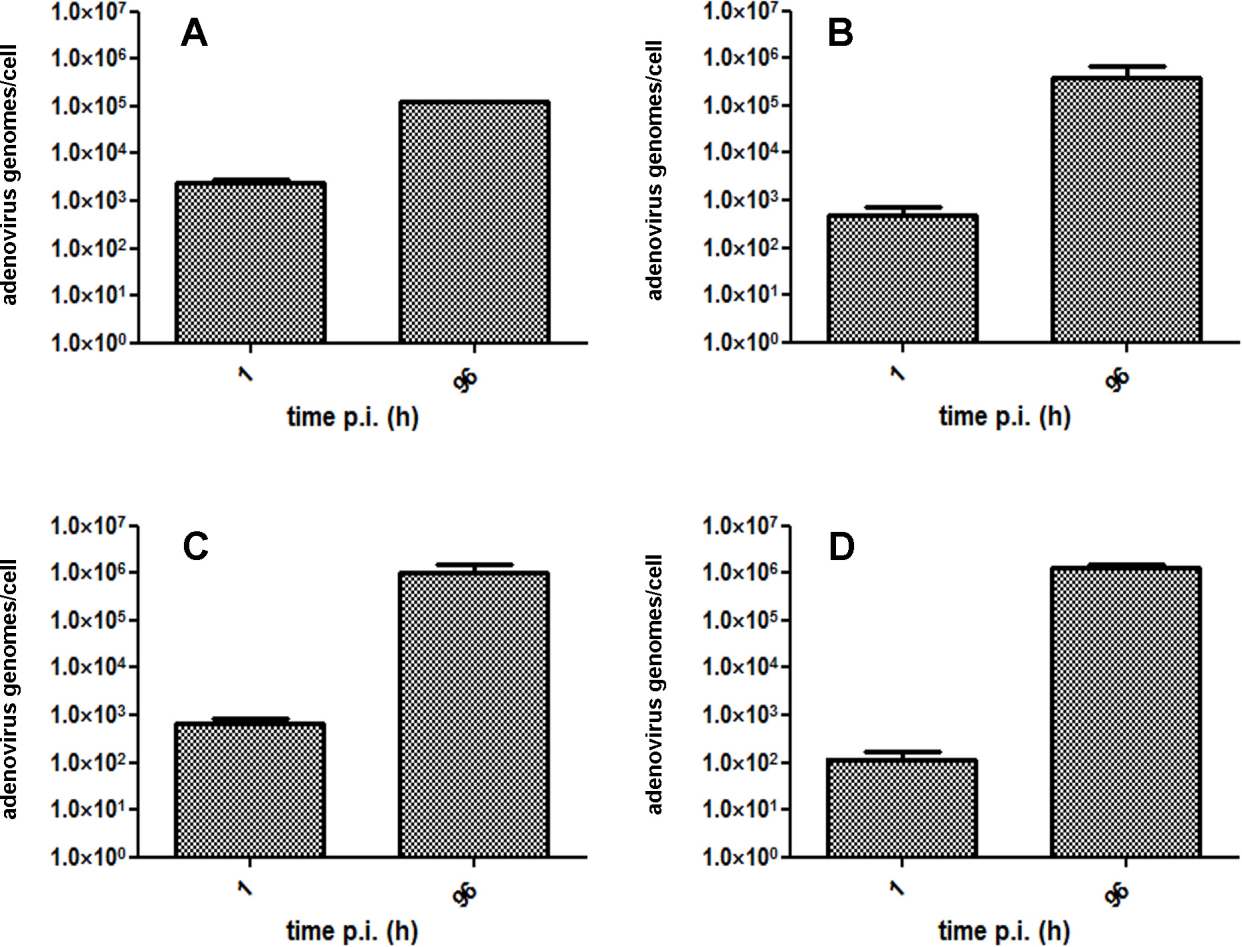
Replication of viral DNA after human adenovirus-D37 infection of corneal cells. Intracellular human adenovirus-D37 (HAdV-D37) DNA concentration (mean adenovirus genomes per cell) at 1 h PI (baseline) and at 96 h PI of primary porcine corneal epithelial cells (PPCE; **A**), primary human corneal epithelial cells (PHCE; **B**), primary porcine corneal keratocytes (PPCK; **C**), and primary human corneal keratocytes (PHCK; **D**). Error bars indicate standard deviation. Increase in HAdV DNA/cell was significant in all cells (p<0.01, unpaired, two-tailed Student *t* test).

The replication of HAdV-D37 DNA was demonstrated in porcine and human corneal cells. Intracellular HAdV-D37 genome copy numbers were quantified with real-time PCR on day 4 post infection (PI) (96 h PI) in comparison to the control samples (baseline HAdV-D37 DNA concentration) obtained 1 h PI. In the epithelial cells, an increase in genome numbers per cell by two logs was observed in PPCE and by three logs in PHCE (Figure 3A,B). HAdV-D37 genome numbers per cell increased significantly (p<0.01, unpaired two-sided Student *t* test) by about three logs in PPCK and by about four logs in PHCK demonstrating HAdV DNA replication in human and porcine cells (Figure 3C,D).

### Porcine corneal cells were fully permissive for human adenovirus infection

**Figure 4:**
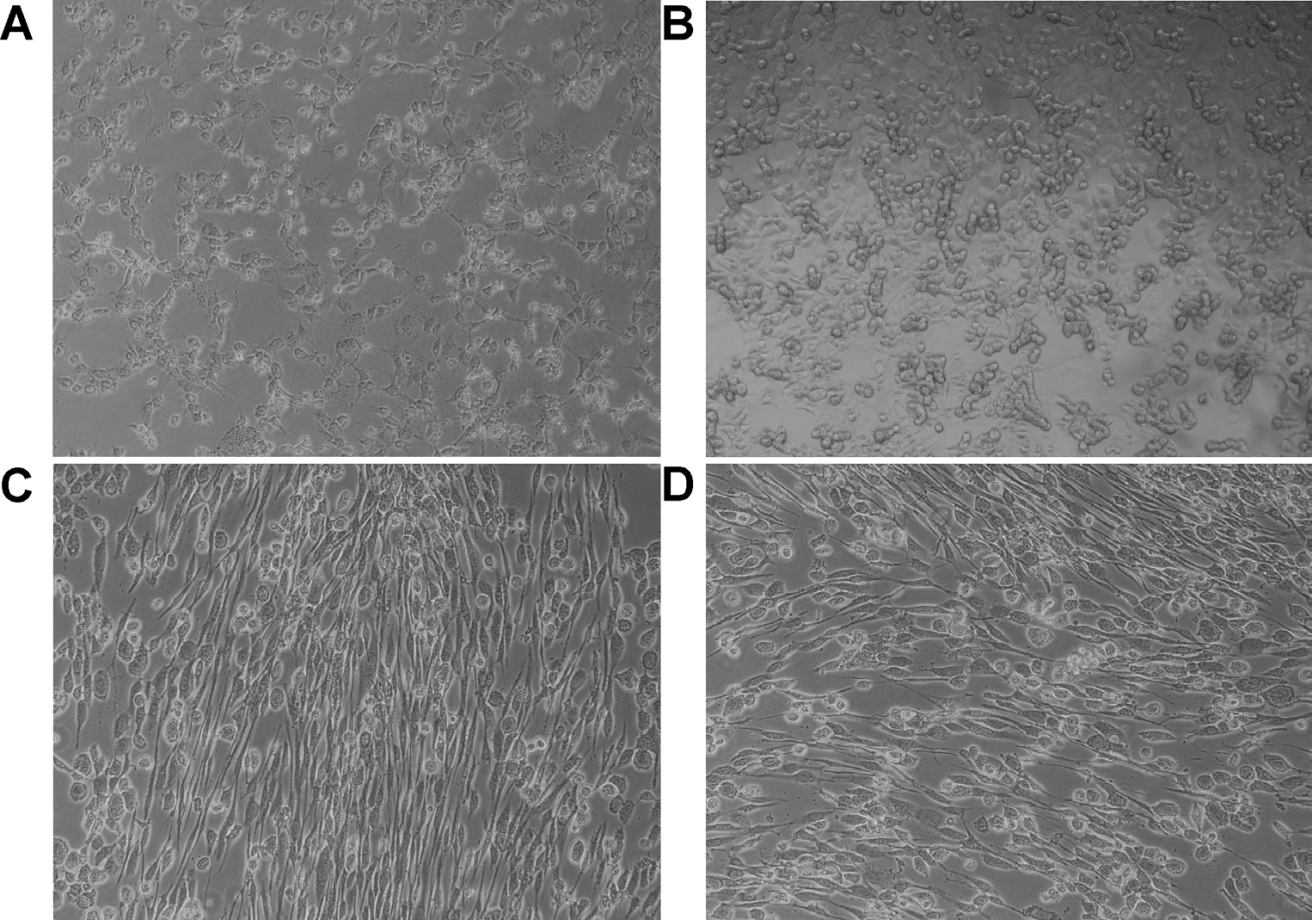
Cytopathic effect caused by human adenovirus-D37. Primary porcine corneal epithelial cells on day 7 PI (**A**), primary human corneal epithelial cells on day 4 PI (**B**), primary porcine corneal keratocytes on day 8 PI (**C**), and in primary human corneal keratocytes on day 5 PI (**D**).

**Figure 5:**
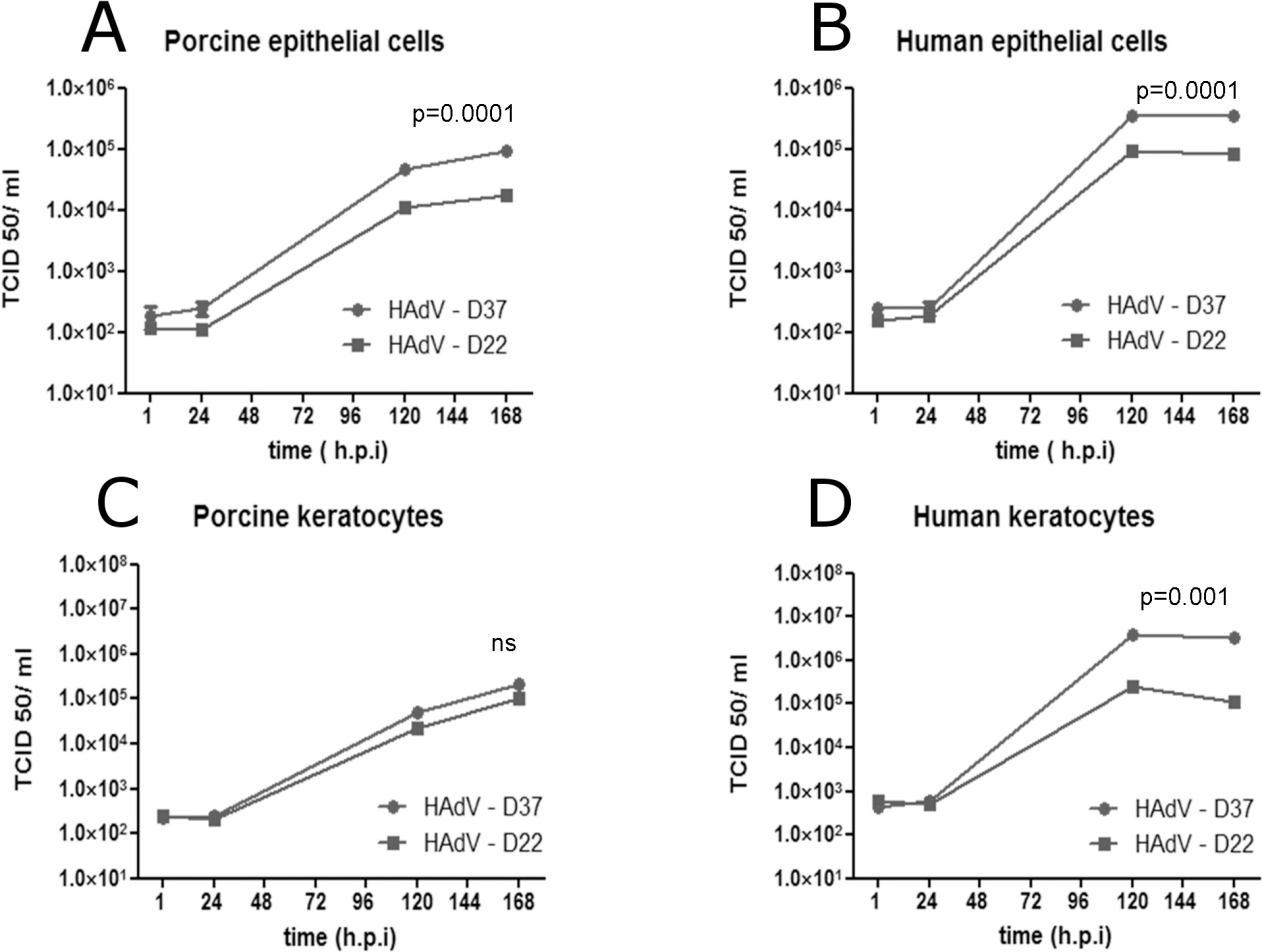
Release of infectious virus progeny to cell culture supernatants. Primary porcine corneal epithelial cells, PPCE (**A**), primary human corneal epithelial cells, PHCE (**B**), primary porcine corneal keratocytes, PPCK (**C**), and primary human corneal keratocytes, PHCK (**D**) after infection with human adenovirus-D37 and -D22, respectively. Calculated p values relate to human adenovirus-D37 (HAdV-D37) and HAdV-D22 virus titers at 168 h PI (unpaired, two-tailed Student *t* test).

PPCE and PPCK were infected with HAdV-D22 and HAdV-D37. PHCE and PHCK were infected in parallel as positive controls. Development of a typical CPE and release of infectious virus progeny were evaluated. After HAdV-D37 infection, CPE was observed on day 7 PI in PPCE (Figure 4A) compared to day 4 PI in PHCE (Figure 4B). In the keratocytes, the CPE was observed on day 8 in PPCK (Figure 4C) compared to day 5 in case of primary human keratocytes (Figure 4D). Release of infectious virus progeny to the culture supernatant was demonstrated on day 5 (120 h) PI and day 7 (168 h) PI In PPCE and PPCK, HAdV titers increased by about two logs from day 1 to day 5 (Figure 5A,C) compared to about three logs in human corneal cells (Figure 5B,D), thus demonstrating the release of infectious virus progeny from infected cells. The HAdV-D37 titers were significantly higher than the HAdV-D22 titers on day 7 PI in PPCE, PHCE, and PHCK but not in PPCK (Figure 5).

### Human adenovirus-D37 induced interleukin-8 mRNA in porcine corneal cells

**Figure 6:**
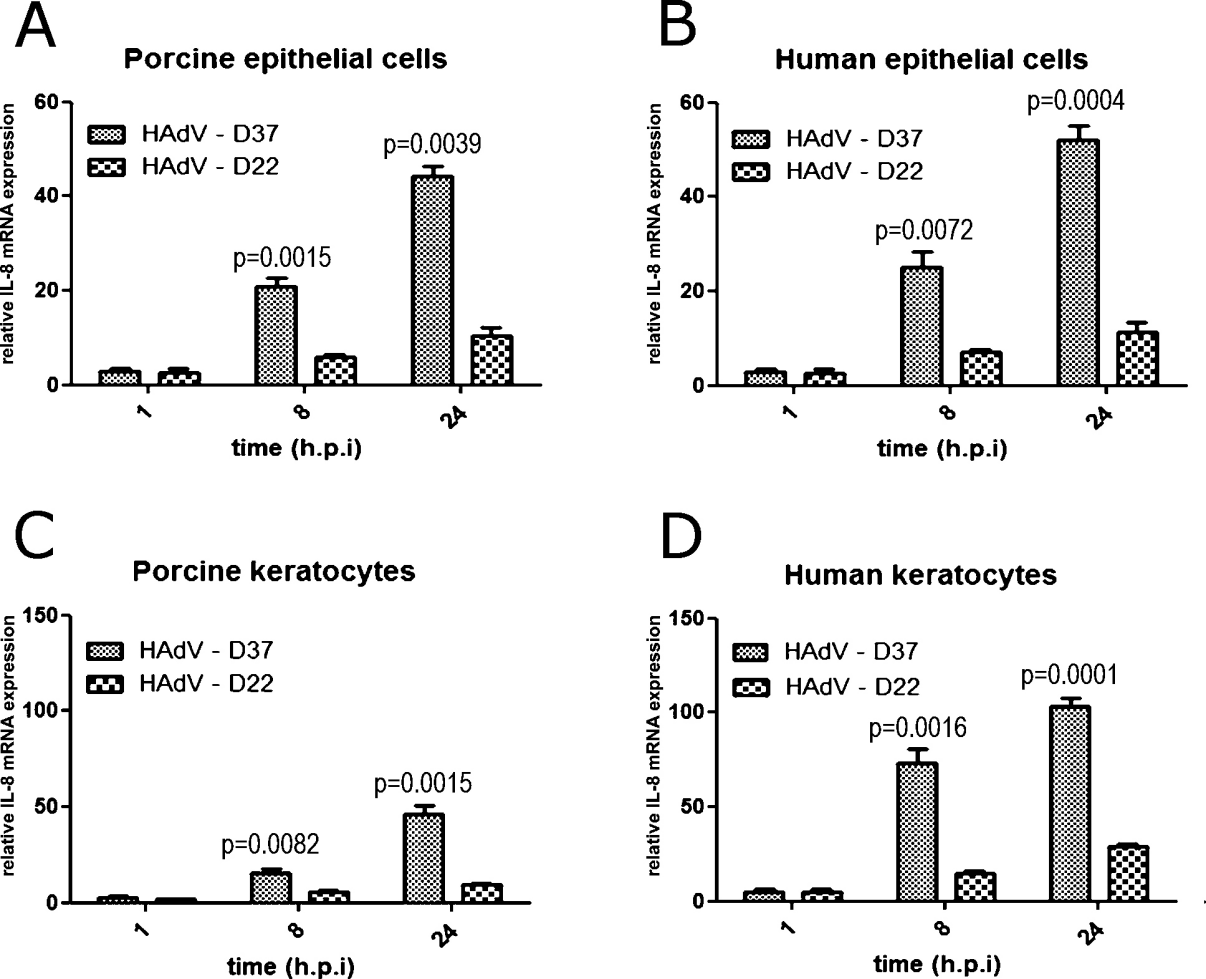
Comparison of interleukin-8 expression on the mRNA level after infection with epidemic keratoconjunctivitis–type human adenovirus-D37 and non epidemic keratoconjunctivitis–type human adenovirus-D22. Primary porcine corneal epithelial cells, PPCE (**A**), primary human corneal epithelial cells PHCE (**B**), primary porcine corneal keratocytes PPCK (**C**), and primary human corneal keratocytes PHCK (**D**), respectively (unpaired, two-tailed Student t test). Increase of interleukin-8 (IL-8) messenger ribonucleic acid (mRNA) concentration was quantified with real-time polymerase chain reaction (PCR) in comparison to mock infected controls.

The induction of IL-8 mRNA by the EKC-type HAdV-D37 was observed as early as 8 h PI in the PPCE and the PPCK as well as in the human cells (PHCE and PHCK). The mRNA levels of IL-8 mRNA were significantly higher (p<0.01, unpaired, two-sided Student *t* test) in the HAdV-D37-infected cells compared to the HAdV-D22-infected cells at 8 h PI (Figure 6). The IL-8 mRNA levels measured at 24 h PI confirmed these results (Figure 6).

## Discussion

In this study, we demonstrated for the first time that HAdV-D37 and HAdV-D22 replicated in primary porcine corneal cells PPCE and PPCK. HAdV-D37 and a few other HAdV types (HAdV-D8, -D19a (-D64), -D53, and -D54) are the etiology of EKC, the only HAdV disease with corneal involvement. In the newly developed porcine cell culture model, HAdV-D37 had a different phenotype regarding IL-8 induction (Figure 6) and replication kinetics (Figure 5) compared to the non-EKC-type HAdV-D22.

Tropism and pathophysiology of EKC-related HAdV types were previously studied in simian virus 40 (SV-40) immortalized human corneal epithelial cells [8,9]. However, recent publications described a modified genomic content and modified gene expression pattern of these immortalized cells compared to primary human corneal epithelial cells [28,29]. Both human cell types (PHCK and PHCE) were permissive for HAdV-D37 replication in vitro confirming results with another EKC-associated HAdV type (HAdV-D19) [30], although HAdV replication in the cornea stroma was never demonstrated in vivo. Unfortunately, to date investigations on this topic have not been published.

The availability of human eye cells as PHCK and PHCE is limited, but recent reports demonstrated that HAdV-C5 can replicate in pigs and cell cultures of porcine origin in spite of its host species specificity [21-23]. No data on the infection of porcine eyes have been published so far. Therefore, we developed porcine cell culture models of corneal HAdV infection: PPCE and PPCK. Interestingly, PPCE and PPCK can be cultured only for shorter periods than human corneal cells.

HAdV-D37 caused a typical CPE in PPCK and PPCE, but development of the CPE was slightly delayed compared to human cells (Figure 2). Furthermore, infection of the PPCE and the PPCK resulted in release of infectious virus progeny to the culture supernatant, but compared to PHCE and PHCK, lower virus titers were observed (Figure 4). These results indicated that porcine corneal cells were fully permissive for HAdV-D37, however virus replication is less effective compared to primary human corneal cells derived of the natural host species of HAdV-D37. In summary, primary human cornea epithelial cells and keratocytes seem to be the preferred model for studying the interaction of HAdV with their target cells, but these cells are not readily available.

Most HAdV types bind with their fiber knob to the cell surface protein CAR as primary receptor [31-33], whereas many, probably all, EKC-causing HAdV types use α2,3-linked sialic acid as the first receptor [8,9]. Obviously, the binding of EKC-type HAdV-D37 to α2,3-linked sialic acid is one reason for the cornea tropism and the ability of these viruses to cause EKC. HAdV-D37 replicated to significantly higher titers than the non-EKC-type HAdV-D22 in human corneal cells (PHCE and PHCK). This difference was also observed in PPCE, but the difference between HAdV-D37 and HAdV-D22 was not significant in PPCK (Figure 5). PPCE expressed higher levels of α2,3-linked sialic acid than PHCE (Figure 2). Therefore, enhanced binding and internalization of HAdV-D37 to PPCE might have compensated the slightly impaired latter steps of the HAdV replication cycle in the porcine cells. In contrast, α2,3-linked sialic acid expression of PPCK was similar to PHCK (Figure 2), and consequently, HAdV- D37 replication was impaired in PPCK compared to the human keratocytes (PHCK, see Figure 5).

Moreover, the EKC-associated HAdV-D37 induced IL-8 mRNA to significantly higher levels than HAdV-D22 in PPCK and PPCE similar to the human cells. Induction of IL-8 was rapid and may be caused by the early steps of the replication cycle as, for example, binding of the virus to the target cell. This hypothesis is supported by the finding that the mouse homolog of IL-8 (“KC,” identical to CXCL-1) was induced in animal model systems [19] that are not permissive for HAdV replication.

Thus, the present study demonstrated for the first time the use of primary porcine corneal epithelial cells and keratocytes for studying EKC-related HAdV types. The results demonstrated that porcine corneal cells are permissive for HAdV-D37 and -D22 infections and that the EKC phenotype of HAdV-D37 can be discerned from the non-EKC phenotype of HAdV-D22. Virulence and tropism determinants of HAdV EKC types may be studied using the porcine cell culture system with the help of recombinant HAdV types. Furthermore, the easy availability of porcine corneal cells make this model attractive for future studies.
